# Caregivers’ experiences of accessing HIV Early Infant Diagnosis (EID) services and its barriers and facilitators, India

**DOI:** 10.1186/s12913-023-10500-z

**Published:** 2024-01-04

**Authors:** Kalyani Nikhare, Nilesh Gawde, Suchit Kamble, Noopur Goel, Sushmita Kamble, Swapna Pawar, Pratik More, Neha Kapoor, Vinita Verma, Bhawani Singh Kushwaha, Chinmoyee Das, Shobini Rajan

**Affiliations:** 1 ICMR - National AIDS Research Institute, Pune, India; 2 Tata Institute of Social Sciences, Mumbai, India; 3 National AIDS Control Organisation, New Delhi, India

**Keywords:** Early infant diagnosis program, Infants exposed to HIV, Caregivers, Barriers and facilitators, India

## Abstract

**Background:**

India has rolled out Early Infant Diagnosis (EID) program for HIV infection in all states. EID program consists of testing of Infants exposed to HIV periodically over 18 months of age which is a multi-step complex testing cascade. Caregivers represent the primary beneficiary of EID program i.e., infants exposed to HIV and face multiple challenges to access EID services. As part of national EID program outcome assessment study, this study narrates caregivers’ perspectives on barriers and facilitators to access and utilize EID services.

**Methods:**

The study was conducted in 31 integrated counselling and testing centres (ICTCs) located in 11 high burden HIV states. A total of 66 in-depth interviews were conducted with caregivers’ of infants enrolled in EID program. Thematic analysis was carried out to help identify themes underlying barriers and facilitators to access EID services and utilization from caregivers’ perspectives.

**Results:**

The stigma and discrimination prevalent in society about HIV remains a key demand side (caregiver-level) barrier. Non-disclosure or selective disclosure of HIV status led to missed or delayed EID tests and delayed HIV diagnosis and initiation of Anti-Retroviral Therapy (ART) for infants exposed to HIV. On supply side (health system-level), accessibility of healthcare facility with EID services was reported as a key barrier. The distance, time and cost were key concerns. Many caregivers faced difficulties to remember the details of complex EID test schedule and relied on a phone call from ICTC counsellor for next due EID test. Delayed EID test results and lack of communication of test results to caregiver were reported as primary barriers for completing the EID test cascade.

**Discussion:**

The study reports caregiver-level and health system-level barriers and facilitators for access to EID services from the caregivers’ perspectives. While, decentralisation and single window approaches can improve the access, timely communication of test results to the caregiver also need to be built in with appropriate use of technology. A holistic intervention including PLHIV support networks and the peer-led support mechanisms would be useful to address societal factors.

**Conclusion:**

The study findings have high significance for developing program implementation strategies to improve access and to build right-based and patient-centred EID services.

## Background

India implemented the Early Infant Diagnosis (EID) program with molecular test-based diagnosis of HIV infection among new born babies born to mothers living with HIV (Infants exposed to HIV) in 2010 [1, 2]. The National AIDS Control Organization (NACO) has rolled out this initiative in a phase-wise manner and it was implemented in all Indian states in phase IV (2012–2017). The India HIV estimate 2021 report showed that around 20,610 pregnant women were estimated to be HIV positive and needed Anti-Retroviral Therapy (ART) treatment for the control of replication of HIV virus [3]. The vertical transmission of HIV has been declining due to the use of anti-retroviral therapy (ART) for the pregnant women and anti-retroviral (ARV) prophylaxis for newborns. Infants born to mothers living with HIV should get exclusive breastfeeds up to 6 months, ARV and Co-trimoxazole prophylaxis, EID testing along with other services like growth and nutrition monitoring, immunisation and other infant care. Under National AIDS Control Program, ARV and Co-trimoxazole prophylaxis along with EID testing services are provided through Integrated Counselling and Testing Centres (ICTCs). However, all the new-borns born to HIV positive pregnant women would need access to EID services. With the EID services, diagnosis and early initiation of ART can be achieved; a step vital for preventing infant deaths due to HIV [2, 4–6].

The HIV testing among infants exposed to HIV include DNA PCR test at 6 weeks and antibody test at 6 months and 12 months. If any of these tests detect presence of HIV or antibody to HIV, additional samples are collected to confirm the diagnosis using DNA PCR tests. Additionally, an antibody-based HIV test is done at 18 months of age. For infants under 6 months, the samples are collected at healthcare facilities with Integrated Counselling and Testing Centre (ICTC) services in the form of dried blood spots (DBS) and transported to six regional reference laboratories (RRLs) where the tests are conducted. For infants above 6 months of age, antibody tests are conducted at the ICTCs and if found to be reactive, the DBS sample is collected at the ICTC for testing at RRL. The antibody-based HIV test at 18 months of age confirms the status of HIV infection. Vertical transmission can occur during infancy due to breast feeding. Therefore, it is critical that all infants exposed to HIV visit ICTCs periodically until the age of 18 months to complete the EID testing cascade for HIV diagnosis and confirmation. The infants need to be diagnosed early and if found positive, they can be initiated on ART treatment [7, 8]. Hence, early infant diagnosis is also a key driver for early initiation of infant ART to reduce mortality and morbidity among HIV-infected infants [9].

Globally, the HIV-AIDS literature has highlighted the challenges of implementing various prevention and treatment programs in resource-limited settings. The global evidence on EID service delivery and uptake highlights the myriad of individual-level (caregiver-level) challenges faced by caregivers and health system-level barriers accessing the services and completing the EID testing cascade in time. These factors were also found to influence or interact with each other in multiple ways; for e.g., long distance to a health facility was compounded by unaffordability, a lack of transport facilities and long waiting time at the clinic [8]. Studies conducted in Indian settings have underscored the shortcomings of the EID program, such as the complex EID testing algorithm for HIV to be followed over 18 months, high rate of loss to follow-up at each step of EID testing cascade, inadequate testing facilities, and lack of trained staff [10–16]. Although these studies identified the programmatic gaps, the analysis was based on recorded data and provider side perspectives, and often were from a single facility or a single city. We approached caregivers from 11 high burden states across the country to understand their perspectives on accessing and utilizing EID services for their infants.

An infant’s engagement in EID services is inextricably linked to that of caregivers. Access to EID services relies heavily on the health-seeking behaviour of caregivers, which in turn is determined by their knowledge of HIV infection and transmission, and their own adherence to ART [7–19]. The published literature from sub-Saharan African settings with a high burden of HIV infection emphasizes the importance of listening to the caregivers’ voices who represent the beneficiaries of the EID programme [9]. To date, there is no study from India that reports the barriers and facilitators accessing EID services from caregivers’ perspectives. Hence, understanding the caregivers’ views would help incorporate community perspective into building knowledge and strengthening rights-based EID services.

## Methods

The study was conducted in 31 ICTCs (healthcare facilities) selected through Probability proportional to size (PPS) sampling from 11 states. A total of 66 in-depth interviews (IDIs) were conducted with caregivers (aged 18 and above) of HIV exposed infants who were enrolled in these centres for EID testing and had undergone at least one EID test from April 2017 to March 2018. The study team prepared the list of caregivers who fulfilled the eligibility criteria by reviewing the registers maintained at the ICTCs. The caregivers were categorized based on age at first EID test. Group A had caregivers who did the first test within first 8 weeks. Group B included caregivers who did the test after 8 weeks and Group C who did not undergo any test. The ICTC counsellors contacted the caregivers, and explained to them the purpose of study, voluntary nature of participation, and invited them for the interview. Most of the Group C caregivers were lost-to-follow-up cases and some refused to participate in the study. The willing caregivers were asked to decide on the place of interview: ICTC or caregivers’ house, or another place as per their preference. All of the willing caregivers (mother, father or grandparents in case of absence of child parents) in this study chose their respective ICTCs for the same. At the ICTC, the counsellors introduced them to the study team. The field investigator introduced the study, read out the participant information sheet and those who consented for participation were interviewed. The written informed consent was translated into local languages. The interviews were guided by an in-depth interview (IDI) guide. The guide explored caregivers’ knowledge regarding EID tests and the test schedule, availability and accessibility of EID services, experiences of visiting ICTCs and counselling for EID tests, difficulties faced to access EID services, reasons for delayed and incomplete EID cascades and dropouts, communication of test results, and suggestions for improvement of EID services.

Data collection and analysis: The study team was trained on qualitative data collection methods, data collection tools, and informed consent procedure. IDIs were conducted in local languages or other languages preferred by participants, and audio was recorded with prior consent from the caregiver. The transcriptions in the local language and translations into English were done through a translation agency. The data were analysed using Atlas Ti Version 6.1.1, a qualitative data analysis software package. The codebook, consisting of codes, sub-codes, and definitions, was developed based on research questions, an interview guide and previous literature. The codes were assigned to representing quotes using Atlas Ti. Any emergent codes and sub-codes were added to the codebook during the coding process. Thematic analysis was carried out to help identify themes underlying barriers and facilitators to accessing and utilizing EID from caregivers’ perspectives.

### Ethics approval and consent to participate

The study was approved by the Institutional Ethics Committee of ICMR-National AIDS Research Institute, Pune (Ref no. NARI/EC/15–16/145) and Institutional Review Board of Tata Institute of Social Sciences, Mumbai (Ref no. TISS/ IRB/19Dec2014), and National AIDS Control Organisation (NACO) ethics committee (Ref no. 02/14/TRG). All research study methods were carried out in accordance with relevant guidelines and regulations. Written informed consent was recorded from all the participants at ICTCs.

## Results

A total of 66 caregivers were interviewed at the 31 selected ICTCs. Mostly, caregivers had basic primary education, were employed in the informal sectors and came from poor socio-economic backgrounds. The stigma and discrimination prevalent in society about HIV remain a key demand side (caregiver-level) barrier whereas accessibility of healthcare facility with EID services is a key supply side (health system-level) barrier. The EID testing cascade is complex and difficult to comprehend for caregivers.

### Stigma and discrimination

Stigma and discrimination were the most common barriers reported by caregivers while accessing HIV services for themselves as well as for the infants exposed to HIV. Disclosure of HIV status was challenging for caregivers, and they feared the consequences of disclosing HIV positive status to other family members, such as victim blaming, disgrace to the family reputation, discrimination, and expulsion or abandonment from family. Fearing the discriminating behaviour by family members, caregivers chose to disclose their HIV status only to close family members who would accept their HIV status and provide support over time. Selective disclosure of HIV status to partner and family members also contributed as a barrier to missed or delayed EID tests and delayed diagnosis and initiation of ART. Caregivers had to make some excuses to visit ICTC centres for EID tests. *“We didn’t tell our family that we have this disease (HIV). I tell them that I am going to hospital because I got fever. We didn’t tell anyone that we have this disease (HIV).”* Counselling of family members by ICTC staff and healthcare provider-assisted disclosure helped in a few cases to gain family support. Caregivers with good family support availed of EID services on time and were motivated to provide the best care for their child. A caregiver shared positive experience, *“We have one son. He is negative. I am negative. Only my wife is positive… In our home, as we are educated, we only take extra care in our life…”*.

Instances of divorce and destitution were also reported, more specifically for discordant couples when only the mother was tested positive and the husband tested negative. Some mothers became solely responsible for their child and they worked and catered to all the needs of their child alone. “*No one supports me; I do (everything) all by myself.” (a mother).* In a few cases, mothers returned to their parents’ house, who took on the responsibility of mother and child. A caregiver (a child’s grandparent) shared, *“her husband gave her divorce when she was 5 months pregnant, after divorce we didn’t know what to do, wanted to abort the child. At this hospital, Madam told us it will be wrong thing to do. So, we took medication and tablets, and HIV test and medication I got it done for her. After that her child was born”.*

The mothers have limited autonomy and mobility and were not allowed to go to ICTC centres for EID tests alone. They were usually accompanied by their husbands or other family members, as they were the ones who primarily took decisions for mother and child. A mother said, *“I can’t come (to ICTC centre) when Ma’am (ICTC staff) called me, my husband would come, I can’t go out of the house.”* In some cases, mothers who faced conflicts at home sought help from front-line workers, such as Accredited Social Health Activist (ASHA worker) to access EID services, a mother shared her experience as, *“I have left home due to lots of problems, like drinking, quarrelling. My family people might hit me, so ASHA worker got me here (ICTC centre)”.* ASHA workers are trained female community health activist selected from the community to work as an interface between the community and the public health system.

Caregivers shared that they cautiously chose not to disclose their HIV status to others outside their family due to fear of discrimination and exclusion. Caregivers preferred not to avail HIV services for themselves or their child fearing that other people would know about their’ HIV positive status when they visit healthcare facility for HIV services. A caregiver shared her experience, *“If anyone comes to know this, they may talk bad, I fear. I cried. Even my mother said the child is not necessary.”* A male caregiver’s view and experience was different, *“Nowadays, this has become normal, there is a lot of awareness about this (HIV) on TV, so now it doesn’t matter what others think.”* He further added that *“I want to look after my child’s future, I don’t care what others think.”* Caregivers were aware of causes of HIV infection and *‘didn’t think much about the society*’. A caregiver shared similar sentiments, *“Some neighbours know about this (HIV status), what can they do? We didn’t get this on our own. Any human can get any disease.”*

### Availability, accessibility and affordability of EID services

The EID testing cascade warrants caregivers to visit ICTCs periodically up to 18 months of Infant’s age for HIV diagnosis and confirmation. Caregivers reported the challenges of traveling long distances to visit the ICTCs and cost of travel as the commonest barriers to access EID services. EID services were available at select ICTCs, and the caregivers living in villages had long distances to cover. The distance, time, and cost were key concerns. *“It (ICTC centre) is too far and too many patients are there, if we go there in the morning our full day is engaged there only. We had to come and go by bus and it was difficult.”* Carrying a young child for the test to another district was also challenging, as one caregiver shared, “*I come from a very long distance around 40 km, that too with 2 children. So, traveling long distance with children is difficult.”* Caregivers also travelled to two differently located health care settings: ART centres to avail ART treatment for the mother and EID services for the infant which resulted in missed or delayed EID tests. Caregivers asked for transfer when eventually EID services were made available at the nearest centre, “*We again came to this centre only as the other centre was far away for us. We cannot travel all the way there carrying baby, hence (we) got transferred here.”*

In many families, the father was ‘sole earning member’ and had low wages and didn’t have any fixed income. They skipped their work and travelled to other district or city by private transport facilities resulting in a decrease in income due to lost wages and an increase in expenses due to travel. Caregivers visited ICTC facilities only when they had enough money to travel to the centre. This often led to missed or delayed EID tests, *“Sometimes money will be there and sometimes it won’t be there, Sir. If money is not there, we will not come, we will see afterward.”* Owing to these factors, some states had initiated travel incentives, but even after completing the necessary administrative processes, caregivers didn’t receive any incentives, “*We get facilities but travel cost and some money for child’s education or for ourselves is not given.”*

### Knowledge of EID tests and schedule

The ICTC counsellors were informing the caregivers about the purpose and benefits of EID testing for the infant. A caregiver shared, *“We need to get them (child) tested, time to time, if they have any problem (HIV infection) then medicines can be started. Otherwise, there is no problem.”* Caregivers were aware that EID tests would help them to know the baby’s status and would help initiate treatment early. But only a few caregivers demonstrated fair knowledge of the EID test schedule, i.e., the number and timing of the tests. The primary source of information for EID tests for caregivers was ICTC staff. But many caregivers faced difficulties recalling the details of this complex EID test schedule and reported varied forms of schedule. A caregiver missed the 12-month test and shared, “They (ICTC staff) said if your child comes as positive, we can start on medication, so they asked to get him tested at 6 weeks, 6 months and 1.5 years. After that all, they have given report as negative.”

### Challenges of missed, delayed and incomplete EID tests

The EID testing cascade is a complex algorithm and the type of test to be conducted depends on the age of the child and result of the previous test. The caregivers frequently mentioned that they brought their child for EID test when they received a phone call from ICTC staff. *“When ma’am (counsellor) calls us, we come for the test.”* Sometimes, caregivers also forgot to bring their child for next due EID test and relied on phone calls from ICTC counsellors. “*Because madam (ICTC staff) called only, we came twice and got tested. First test was done at 3 months. We forgot about the sixth month test. Madam called again at 6 months, so we came and got tested.”* Following the complex algorithm was difficult for caregivers and they would visit when called for. Active tracking and follow-up by ICTC staff helped to link children exposed to HIV to EID services and visit ICTC centres when the EID test was due.

The duration from sample collection to communication of EID test results to caregivers varied across the states, and caregivers reported that they received the test results as early as 1 month to as late as 6 months after the EID test. In some facilities, caregivers were informed that the samples were sent to a national level laboratory which would take more time for results and they would receive a call for the results. *“It was clearly told us that it will take up to 3 months minimum to get the result. So, be patient and wait for our (ICTC’s) call.”*

The delay in communication of the test results was a significant issue reported by the caregivers which made them less motivated to undergo subsequent tests. Some caregivers said that they received calls from the counsellor asking them to visit ICTC to collect reports when ICTCs received it. But this was not the case for other caregivers who visited the ICTC centre many times for test results. *“Sir, my 45 days’ reports took 4–5 months to arrive and the 2nd test which was for 6th month, till now the report has not come and it’s been 6 months now. I ask them repeatedly about reports whenever I visit here and I get the same answer that report has not come.”* Sometimes caregivers received the result when they visited the centre for the subsequent test. *“When I had come here for a 6-month test that time they gave me the report. That time only I asked them whether the report has come and they said yes.”*

Delayed EID test results and a lack of communication of test results to caregiver were reported as primary barriers to completing the EID test cascade. Delayed test results caused missed tests, delays in subsequent EID tests, and served as a disincentive for caregivers to complete the EID test cascade.

## Discussion

This qualitative study reports the barriers and facilitators to access EID services from caregivers’ perspectives. The caregivers mainly reported the barriers and facilitators experienced at the individual level (caregiver level) and the facility level (health system level). Caregivers faced difficulties accessing EID services because of long distance travel and lack of transport money. This was because EID services were available at select facilities and decentralisation to more facilities would help. The EID tests and mothers’ HIV care services were not timed to align, because mothers visited ART (treatment) centres for their own HIV care and need to visit ICTC (diagnostic) centres for EID services for their new born babies. At times, both are co-located but not necessarily so. Other services for newborn such as vaccinations are part of maternal and child program but these are not offered in integrated manner. Vaccinations are offered in the most decentralised manner at sub-centre and villages, EID services at select Primary Health Centres and ART services at select secondary and tertiary facilities. A Single window approach where HIV care and diagnostic services will be provided under one roof has been recommended and shall be implemented soon. This approach will help caregivers access EID services for their children and ART services for themselves under a single roof. Decentralisation of ART services would also be useful. These findings are coherent with findings from other low- and middle-income countries (LMICs) and Sub-Saharan African settings [8, 9, 17–19]. Another approach can be to collect DBS samples at the nearest health facility or at home as per the caregiver’s consent.

Further, the caregivers who brought their child for EID tests faced another set of challenges. The confirmation of HIV infection is based on antibody test at 18 months of age. The infant has to be brought to the facility at least four times, i.e., at 6 weeks, 6 months, 12 months, and 18 months, and more, if any test result is positive. The EID test algorithm is a complex, multi-step process, and caregivers often missed their child’s test visit. Our EID program outcome assessment study showed that only 78% of the infants exposed to HIV had at least one EID test. Of the infants who had HIV tests, 50% had at first sample collected by 8 weeks of age [20]. A study conducted in rural Uganda showed that only 11% caregivers were fully aware of the frequency and proper scheduling of EID services and follow-up of missed appointments helped to improve EID service utilization [21].

The ICTC counsellors were the primary source of information for caregivers. Caregivers relied on phone calls from ICTC staff for information on the next due test. HIV counselling, active tracking of mothers living with HIV and their infants and regular capacity building sessions for ICTC staff will facilitate EID service utilization and completion of EID testing cascade. A cluster-randomised trial in Maharashtra, India demonstrated that the use of mHealth-facilitated behavioural training intervention for outreach workers increased uptake of early infant diagnosis services. Such capacity building activities for the EID program will positively impact service delivery and utilization by caregivers [22].

Previous studies have shown that a long turn-around-time for the receipt of DNA-PCR test results, inconsistencies in results, failure to convey HIV test results to caregivers and a lack of infant exposed to HIV tracking and follow-up systems led to the non-completion of EID testing cascade and a high loss to follow-up [7, 18, 19, 23]. The regional reference laboratories share the test results with ICTC centres and ICTC centres share the results with caregivers. The caregivers reported waiting for the test results even for 6 months and there was no systematic mechanism in place for communication of test results to them. The long turn-around-time caused delayed or missed EID tests and the lack of communication also contributed to loss-to-follow-up. Analysis of national-level data from regional reference laboratories in India demonstrated that the time lag between sample collection and sample testing positively contributed to delayed HIV diagnosis [24]. The delay in communication of results to caregivers even when the test results were available adds insult to the injury. This needs to be avoided. In Kenya, an online system - HIV Infant Tracking System (HITSystem) used algorithm-based computer alerts for EID laboratory staff, and text messaging alerts for mothers to improve enrolment and retention in EID testing cascade [25]. India adopted the system-generated email communication to reduce time to communicate positive test result to ICTC so that subsequent samples for confirmation of diagnosis can be collected or child can initiate ART immediately. The delays from ICTC to the caregiver needs to be looked into and the all reports can be shared online with the facilities.

Stigma and discrimination were the underlying factors that delayed access to the EID services. Stigma made the caregiver share their HIV status only with a few persons within the family. They had to think about reasons to share with family members when they had to visit a distant town with the infant for testing. If their HIV status was known to the family members, the support they received from husband and family determined the access to EID services for the infants exposed to HIV (20). Destitution was common irrespective of the HIV status of the husband, but women with HIV negative husband faced additional brunt about fidelity and morality. Previous literature also highlights mother’s non-acceptance of her HIV status, fear of disclosure of maternal HIV status, lack of partner and social support, fear of HIV-positive result for the baby, and cultural factors as social barriers to access EID services [1, 8, 9, 17, 21–26].

The stigma and discrimination seem to operate differently for men and women. The quotes from fathers (irrespective of their HIV status) indicate much less stigma and discrimination experienced by them and they were not constrained by societal values and had autonomy of mobility. Women however faced more internal stigma, overt discrimination including destitution and abandonment. Women reported much less support from both family and society and had limited autonomy of mobility. Infant care being the primary responsibility of women also affected the access of the HIV-exposed infant to EID services. Involvement of male partner in HIV care, and social support are among the key facilitators of EID service uptake [19, 27, 28].

There were a few limitations associated with this study. The study participants were the caregivers who had their child tested at the selected ICTCs. The lack of caregiver details (phone numbers and address) posed a challenge to select caregivers based on caregiver type (mother, father, grandparents), and caregiver HIV status. The caregivers who never accessed the EID services and the caregivers who dropped out of services could not be retrieved by counsellors, as these were the loss-to-follow-up cases. The study could not capture the reasons for not accessing the EID services. Also, the number of caregivers declined to participate in study and their socio-demographic details were not captured. Caregivers also accessed HIV services for themselves at ART centres and the study did not explore the role of ART counsellors in sensitising caregivers for importance of and improving uptake of EID testing services. However, the purpose of this paper was to understand barriers and facilitators and the system-level experiences can be best articulated by those who used the system. Also, the challenges reported in this study still persist even 5 years after conduction of these IDIs and there is no recent data available to improve the program implementation [20, 24].

In conclusion, the study reports caregiver-level and health system-level barriers and facilitators for access to EID services from the caregivers’ perspective. While decentralisation and single window approaches can improve access, timely communication of results to the caregiver also needs to be built in with appropriate use of technology. Apart from these two factors, stigma and discrimination and the status of women in society were key barriers. A more holistic intervention would be needed for the same. The PLHIV are already part of care and support networks, and the peer-led support mechanisms may be very useful in addressing some of these societal factors. The study findings have high significance for developing program implementation strategies to improve access and build right-based and patient-centred EID services.

## Data Availability

The data used and/or analysed during the current study are available from the corresponding author on reasonable request.

## Abbreviations

ART: Anti-Retroviral Therapy
ARV: Anti-retroviral
ASHA: Accredited social health activist
DBS: Dried Blood Spot
EID: Early Infant Diagnosis
ICTC: Integrated counselling and testing centre
IDI: In-depth interviews
LMIC: Low- and Middle-Income Countries
NACO: National AIDS Control Organization
PLHIV: People living with HIV
RRL: Regional Reference Laboratory
